# Using 30-day modified rankin scale score to predict 90-day score in patients with intracranial hemorrhage: Derivation and validation of prediction model

**DOI:** 10.1371/journal.pone.0303757

**Published:** 2024-05-21

**Authors:** William L. Baker, Mukul Sharma, Alexander Cohen, Mario Ouwens, Mary J. Christoph, Bruce Koch, Timothy E. Moore, Garrett Frady, Craig I. Coleman

**Affiliations:** 1 University of Connecticut School of Pharmacy, Storrs, CT, United States of America; 2 Evidence-Based Practice Center, Hartford Hospital, Hartford, CT, United States of America; 3 Division of Neurology, Population Health Research Institute, McMaster University, Hamilton, Ontario, Canada; 4 Guy’s and St. Thomas’ Hospitals, King’s College London, London, United Kingdom; 5 Medical and Payer Evidence, BioPharmaceuticals Medical, AstraZeneca, Cambridge, United Kingdom; 6 AstraZeneca Pharmaceuticals, Wilmington, DE, United States of America; 7 Statistical Consulting Services, Center for Open Research Resources & Equipment, University of Connecticut, Storrs, CT, United States of America; 8 Department of Statistics, University of Connecticut, Storrs, CT, United States of America; Bach Mai Hospital, VIET NAM

## Abstract

Whether 30-day modified Rankin Scale (mRS) scores can predict 90-day scores is unclear. This study derived and validated a model to predict ordinal 90-day mRS score in an intracerebral hemorrhage (ICH) population using 30-day mRS values and routinely available baseline variables. Adults enrolled in the Antihypertensive Treatment of Acute Cerebral Hemorrhage-2 (ATACH-2) trial between May 2011 and September 2015 with acute ICH, who were alive at 30 days and had mRS scores reported at both 30 and 90 days were included in this post-hoc analysis. A proportional odds regression model for predicting ordinal 90-day mRS scores was developed and internally validated using bootstrapping. Variables in the model included: mRS score at 30 days, age (years), hematoma volume (cm^3^), hematoma location (deep [basal ganglia, thalamus], lobar, or infratentorial), presence of intraventricular hemorrhage (IVH), baseline Glasgow Coma Scale (GCS) score, and National Institutes of Health Stroke Scale (NIHSS) score at randomization. We assessed model fit, calibration, discrimination, and agreement (ordinal, dichotomized functional independence), and EuroQol-5D ([EQ-5D] utility weighted) between predicted and observed 90-day mRS. A total of 898/1000 participants were included. Following bootstrap internal validation, our model (calibration slope = 0.967) had an optimism-corrected c-index of 0.884 (95% CI = 0.873–0.896) and R^2^ = 0.712 for 90-day mRS score. The weighted ĸ for agreement between observed and predicted ordinal 90-day mRS score was 0.811 (95% CI = 0.787–0.834). Agreement between observed and predicted functional independence (mRS score of 0–2) at 90 days was 74.3% (95% CI = 69.9–78.7%). The mean ± SD absolute difference between predicted and observed EQ-5D–weighted mRS score was negligible (0.005 ± 0.145). This tool allows practitioners and researchers to utilize clinically available information along with the mRS score 30 days after ICH to reliably predict the mRS score at 90 days.

## Introduction

Post-stroke functional outcome assessment in individuals with intracerebral hemorrhage (ICH) should consider the time course of recovery [1,2]. Functional recovery evolves over months, and early assessments may underestimate final functional outcome. Conversely, the true impact of the index stroke is more difficult to assess as competing causes of morbidity and mortality may occur over time, as may differences in how morbidity is addressed [3]. Factors contributing to this difficulty can include, but are not limited to, quality of rehabilitation, social support, and geographic location, among others. While 90-day modified Rankin Scale (mRS) score after stroke (ischemic or hemorrhagic) has become the standard for primary outcome measurement in acute stroke trials [4], shorter time assessment periods, such as at discharge or 30-days post stroke, are more feasible to capture. To evaluate if 30-day mRS scores can serve as a proxy for 90-day mRS scores, we developed and internally validated a model for predicting 90-day mRS score in ICH patients using their 30-day mRS value augmented by routinely collected short-term clinical data. This tool could utilize clinically available information along with the mRS score 30 days after an ICH to predict the mRS score at 90 days, having both research and clinical applications.

## Patients and methods

This report was written in accordance with the Transparent Reporting of a multivariable prediction model for Individual Prognosis Or Diagnosis (TRIPOD) statement [5].

### Source of data

This is a post hoc analysis of the randomized, controlled Antihypertensive Treatment of Acute Cerebral Hemorrhage II (ATACH-2) trial (ClinicalTrials.gov Identifier: NCT01176565) [6]. ATACH-2 randomized participants from May 2011 through September 2015 with acute ICH (excluding those with a hematoma volume > 60 cm^3^) to either an intensive blood pressure–lowering strategy (goal systolic blood pressure [SBP] of 110–139 mmHg) or a standard blood pressure–lowering strategy (goal SBP of 140–179 mm Hg), started within 4.5 hours of symptom onset and continued for 24 hours. The primary outcome was a composite of death or disability per mRS score at 90 days after randomization. The data used for this analysis was obtained through the National Institute of Neurologic Disorders and Stroke (NINDS) and was provided in a deidentified format. This observational study was performed in accordance with ethical principles consistent with the Declaration of Helsinki, International Conference on Harmonisation Good Clinical Practice, Good Pharmacoepidemiology Practice, and the applicable legislation on noninterventional and/or observational studies. As it complies with the Health Insurance Portability and Accountability Act of 1996, this study is exempt from Institution Review Board oversight per the NINDS.

This analysis included ATACH-2 trial participants who were alive at 30 days after randomization and had an mRS score reported at both 30 and 90 days (including those who died between 30 and 90 days, who were categorized as having an mRS score of 6 at 90 days).

### Sample size

Sample size for this project was estimated through a 2-step process using the methods of Riley and colleagues [7]. We assumed the outcome of mRS score at 90 days to be continuous with a mean value of 2.6 and a standard deviation (SD) of 1.4, an anticipated Nagelkerke’s R^2^ value of 0.6 based on prediction models in an ischemic stroke population [8], and 13 candidate predictor parameters. This suggested a minimum sample size of 247 individuals. We then calculated the “relative efficiency” (relative efficiency of a proportional odds test on the variable relative to the same test on a variable that has no ties) of modeling 90-day mRS compared with a continuous response, resulting in a value of 0.955 [9]. Dividing the estimated sample size (247) by the efficiency (0.955) yields an effective required sample size of 259.

### Outcome and predictors

The dependent variable for the model was ordinal mRS score at 90 days post randomization. Predictor variables included in the model were mRS score at 30 days, age (years), hematoma volume (cm^3^), hematoma location (deep [basal ganglia, thalamus], lobar, or infratentorial), presence of intraventricular hemorrhage (IVH), baseline Glasgow Coma Scale (GCS) score, and National Institutes of Health Stroke Scale (NIHSS) score at randomization. Our dataset had no missing data for outcome or predictor variables.

### Model development and validation

The model was constructed using a cumulative logit model (also called the proportional odds model) in which the outcome of mRS score at 90 days was modeled as an ordinal variable. This model estimates intercepts for each level of the outcome, but assumes a common coefficient across ordered response categories. The validity of the proportional odds assumption was verified by plotting partial residuals.

Overall model fit was assessed using Nagelkerke’s R^2^, which is used to assess goodness of fit and ranges from 0 to 100%, and Brier score, which is a method of verifying predictive accuracy and ranges from 0 to 0.25, where lower values are better. Internal validation of the model was performed using bootstrap resampling (500 times), drawn with replacement, to evaluate the discrimination and calibration of the model and estimate optimism-corrected performance measures [10]. Model discrimination (how well the model separated between individuals who had the outcome and those that did not) was evaluated using the c-index (which corresponds to a nonparametric estimator of the area under the curve), where a value of 1.0 represents perfect model discrimination and a value of 0.5 suggests prediction no better than random chance [11,12]. Model calibration was visually assessed using a calibration plot and calibration slope [13]. A perfectly calibrated model would show an intercept of 0 and a calibration slope of 1. To account for potential overfitting, uniform (heuristic) shrinkage was applied to the final model beta coefficients [14]. This shrinkage factor is an estimate of the in-sample optimism of the calibration slope, as calculated using a bootstrap internal validation. All model analyses were performed in rms R package (version 6.3–0 [15]) with R version 4.2.2 [16] within RStudio version 2022.12.0.

The optimism-corrected regression coefficients from our model were converted to predicted probabilities. The mRS score at 90 days with the highest predicted probability was then identified. Agreement between the predicted mRS score at 90 days and the actual mRS score for each individual ATACH-2 participant in the cohort was assessed through calculation of ĸ using quadratic weights. We also identified the proportion of predicted mRS scores at 90 days that matched or were within 1 unit of the actual mRS scores.

Predicted probabilities of mRS scores at 90 days were also summed to identify those who achieved functional independence, defined as an mRS score of 0 to 2, and those who did not, defined as an mRS score of 3 to 6, based on prior research [17,18]. Absolute agreement between predicted and observed functional independence at 90 days was reported. The sensitivity of this definition was evaluated by assigning functional independence either as an mRS score at 90 days of 0 to 1 (vs 2–6, categorized as dependent) and 0 to 3 (vs 4–6, categorized as dependent).

As utility-weighted mRS scores are increasingly being used as outcome measurements in stroke trials [19], we compared the observed 90-day EuroQol-5D (EQ-5D)–weighted mRS scores to the predicted 90-day EQ-5D–weighted mRS scores. To do so, mean EQ-5D-3L health utility scores reported in ATACH-2 were determined for each mRS score category and assigned for each trial participant based on their observed and predicted mRS score at 90 days (**S1 Table in S1 Appendix**). The mean ± SD differences between participants’ observed and predicted values were calculated.

## Results

A total of 898 of the 1000 (89.8%) participants enrolled in the ATACH-2 trial met inclusion criteria (a sample greater than the estimated required sample size of 259). Characteristics of this cohort are shown in **Table 1**. The median (25^th^, 75^th^ percentile) age was 61 (52, 75) years, with most being men (62.4%) with a baseline SBP of 174 (155, 190) mm Hg, and normal kidney function. For past medical history, a majority (52.2%) were smokers, 18.3% had prior diabetes, 15.1% had a prior stroke, 2.8% had atrial fibrillation, and 24.7% had hyperlipidemia. The median hematoma volume was 9.7 (4.5, 18.9) cm^3^, with 88.9% being deep (56.1% in the basal ganglia, 32.7% in the thalamus), 10.9% lobar, and 0.2% infratentorial. The median baseline GCS score was 15 (13, 15), the median baseline NIHSS score was 10 (6, 15), and about one-half (52.7%) of patients were discharged from the hospital to a location other than home. The median mRS score at 30 days post randomization was 4 (2, 4), with the most common score being 4 (36.9%). Approximately half of patients maintained the same mRS score between 30 and 90 days (**S1 Fig**).

**Table 1:** Baseline characteristics of study cohort.

Characteristic, n (%) or median (25^th^, 75^th^ percentile)	
Age, y	61 (52, 75)
Male sex	560 (62.4)
Race	
White	9 (1)
Black or African American	4 (0.5)
Asian	228 (25.4)
Other	113 (12.6)
Unknown/not reported	544 (60.6)
Baseline SBP, mm Hg	174 (155, 190)
Baseline SCr, mg/dL	0.85 (0.70, 1.10)
Past medical history	
Diabetes mellitus	164 (18.3)
Smoking	469 (52.2)
Stroke	136 (15.1)
Atrial fibrillation	25 (2.8)
Ischemic heart disease	36 (4)
Peripheral vascular disease	12 (1.3)
Hyperlipidemia	210 (24.7)
Mechanical ventilation at randomization	97 (10.8)
Time from symptom onset to randomization, minutes	183 (137, 235)
Hematoma volume, cm^3^	9.7 (4.5, 18.9)
Hematoma location	
Deep (basal ganglia, thalamus)	798 (88.9)
Lobar	98 (10.9)
Infratentorial	2 (0.2)
IVH	238 (26.5)
Hospital length of stay, days	16 (8, 27)
Baseline GCS score	15 (13, 15)
Baseline NIHSS score	10 (6, 15)
Discharge destination at 30 days	
Home	221 (24.6)
Rehabilitation	473 (52.7)
Other	204 (22.7)

GCS = Glasgow Coma Scale, IVH = intraventricular hemorrhage, NIHSS = National Institutes of Health Stroke Scale, SBP = systolic blood pressure, SCr = serum creatinine.

**Fig 1:**
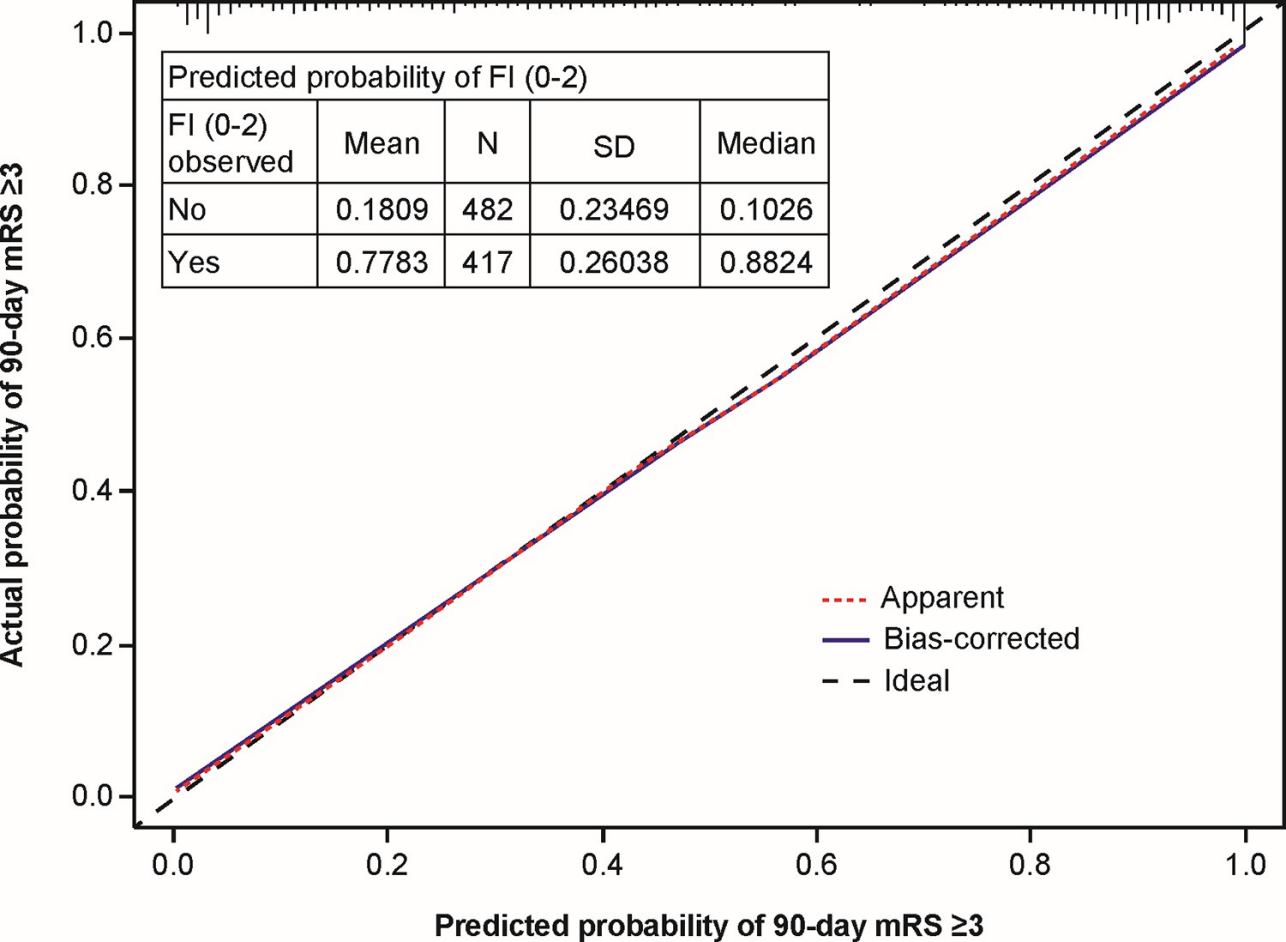
Internal validation calibration plot. Plot shows the calibration of the model comparing the predicted probability of having an mRS score at 90 days of 3 to 6 (x-axis) compared with the actual probability (y axis). The distribution of predicted probabilities is shown across the top axis. Fl = functional independence, mRS = modified Rankin Scale, SD = standard deviation.

The ordinal regression prediction model is shown in **Table 2**, including coefficients for the initial model and the shrunken model (after bootstrapping). The Nagelkerke’s R^2^ and Brier scores for the model were 0.712 and 0.101, respectively, while the c-index for the initial model was 0.886. After internal validation, the optimism-corrected c-index was 0.884 (95% CI = 0.873–0.896) and the calibration slope was 0.967. The calibration plot (**Fig 1**) showed good agreement between the predicted and observed probability of having a 90-day mRS score of ≥3 across the risk range. After applying the uniform shrinkage factor of 0.967 (bootstrapped calibration slope), the final (shrunken) prediction model and coefficients are shown in **Table 2**. Visual evaluation of the partial residual plots supported upholding the proportional odds assumption of the model. A Microsoft Excel–based calculator to generate predicted 90-day mRS scores using the abovementioned model is available in the Supplemental materials.

**Table 2:** Final ordinal regression prediction model.

Variable	Unadjusted model β coefficient (SE)	Adjusted model (shrinkage factor = 0.967) β coefficient
Intercept, mRS at 90 days = 1	–3.872 (0.862)	–3.743
Intercept, mRS at 90 days = 2	–6.885 (0.894)	–6.656
Intercept, mRS at 90 days = 3	–8.862 (0.911)	–8.567
Intercept, mRS at 90 days = 4	–10.725 (0.926)	–10.367
Intercept, mRS at 90 days = 5	–14.167 (0.970)	–13.695
Intercept, mRS at 90 days = 6	–16.222 (1.021)	–15.681
30-Day mRS = 1	1.755 (0.391)	1.697
30-Day mRS = 2	3.205 (0.416)	3.098
30-Day mRS = 3	4.186 (0.429)	4.047
30-Day mRS = 4	6.728(0.446)	6.504
30-Day mRS = 5	8.983 (0.514)	8.683
Age, per year	0.030 (0.006)	0.029
Baseline NIHSS score	0.070 (0.015)	0.068
Baseline GCS score	0.095 (0.045)	0.092
IVH	0.387 (0.158)	0.374
Hematoma volume, per 10 mL	0.033 (0.064)	0.032
Hematoma location = lobar	–0.451 (0.230)	–0.436
Hematoma location = infratentorial	–1.950 (1.438)	–1.885

GCS = Glasgow Coma Scale, ICH = intracranial hemorrhage, IVH = intraventricular hemorrhage, mRS = modified Rankin Scale, NIHSS = National Institutes of Health Stroke Scale, SE = standard error.

**Fig 2:**
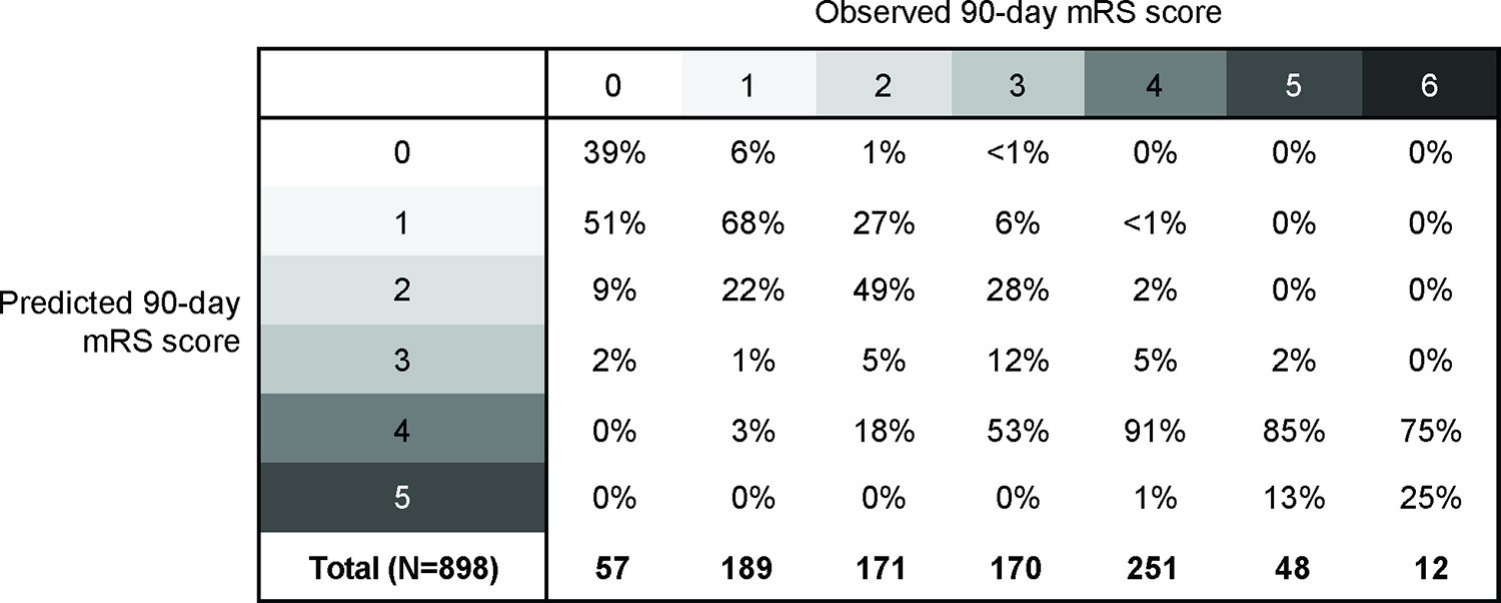
Percent agreement between observed and predicted 90-day mRS score. Percentages total to 100% (or near due to rounding) within each observed 90-day column. mRS = modified Rankin Scale.

**Fig 2** shows the relationship between the observed mRS score at 90 days and the predicted mRS score at 90 days with this model. There was substantial agreement between values, with a weighted ĸ of 0.811 (95% CI = 0.787–0.834).[20] The predicted mRS score at 90 days was within ±1 of the observed value in 91.6% of ATACH-2 participants.

Absolute agreement between observed and predicted functional independence (mRS score of 0–2) at 90 days was 87.2% (95% CI = 84.8%-89.3%) (**Table 3**). Sensitivity analyses defining functional independence as an mRS score of 0 to 1 and 0 to 3 (vs. 0–2) are reported in **S2 and S3 Tables in S1 Appendix**. Agreement was generally higher when using various dichotomization threshold values to define functional independence compared to modeling agreement of exact ordinal scores.

**Table 3:** Differences between observed versus predicted functional independence (mRS Score 0–2 at 90 Days)*.

		Functional independence (0–2) predicted		Total
		No	Yes	
Functional independence (0–2) observed	No	415	66	481
	Yes	49	368	417
Total		464	434	898

mRS = modified Rankin Scale.

*Absolute agreement: 87.2%, 95% CI = 84.8%-89.3%.

The mean ± SD absolute difference in the health utility-weighted mRS score between the predicted and observed 90-day time points was 0.005 ± 0.145.

## Discussion

In patients with acute ICH, we developed and validated a model to predict the 90-day mRS score using 30-day scores in addition to other routinely available variables. The model was shown to reliably predict mRS score at 90 days on an ordinal and as an EQ-5D weighted (utility weighted) scale. Moreover, it could predict functional independence at 90 days. Agreement was related to the mRS score itself (and potentially the clinician’s ability to distinguish between scores), with much of the disagreement between predicted and observed mRS scores at 90 days deriving from individuals who had scores in the midrange at 30 or 90 days. The increased discrepancy between predicted and observed mRS scores in the midrange is in alignment with the interrater reliability expected for clinical assessors of the mRS score [21]. Studies have shown the greatest interobserver variability between mRS scores in the midrange, likely explained by better definitions of outcomes at the highest and lowest categories [22].

We believe our findings are biologically plausible for several reasons. First, this result aligns with prior research in ischemic stroke patients showing that 30-day mRS scores were strongly associated with 90-day scores (Ovbiagele et al. Neurology 2010) [23]. Further, the additional variables such as GCS and NIHSS scores that were chosen as covariates were selected based on prior research showing that they were predictive of functional independence at 90-days. We find it highly plausible that 30-day mRS in conjunction with additional variables are predictive of 90-day mRS, though there are still outstanding research questions related to when functional status should be measured (e.g. 90 days or 6 months), and how to best support functional recovery.

The variables used to develop our model are routinely available to clinicians caring for ICH patients, particularly in geographic areas with coordinated rehabilitation schedules and good access to higher level stroke care [24]. Factors, such as the individual’s age, hematoma volume and location, baseline NIHSS and GCS scores, and IVH are commonly collected during hospitalization for ICH. We have shown that adding these factors to the mRS score at 30 days results in a model with strong calibration and discrimination for predicting the 90-day outcome. Clinicians can utilize the model to determine the predicted probability of an mRS score at 90 days on an ordinal scale, as well as use it to predict functional independence. This can be helpful in tailoring care for individuals, as the economic burden associated with care of stroke patients with poor functional status per the mRS at hospital discharge is greater than for those with greater functional independence [25]. Likewise, researchers can apply the model to data where the mRS score at 30 days is available to predict the 90-day score.

In addition to the 30-day mRS score, covariates chosen for the predictive model were taken from prior scoring tools used to predict death or disability in patients experiencing ICH [25–28]. The ICH score utilized age (<80 or ≥80 years), GCS score, ICH volume and location, and presence of IVH, but was developed to predict 30-day mortality [26]. The subsequently published FUNC score utilized age (<70, 70–79, ≥80 years), ICH volume and location, GCS score, and presence of pre-ICH cognitive impairment to develop a prediction tool for functional status (defined as an mRS score of ≥4 at 90 days) [27]. Using a split-sample method, the FUNC score had good calibration in the development cohort (c-index = 0.88) that somewhat decreased in the validation cohort (c-index = 0.82). The ICH Functional Outcome Score (ICH-FOS) utilized age (continuous), admission NIHSS score, GCS score, admission blood glucose level, ICH volume and location, and intraventricular extension to predict poor functional outcome at 1 year (defined as mRS score of ≥3), although 90-day outcomes were also reported [28]. The ICH-FOS score had a c-index of 0.842 for predicting functional independence 90 days after ICH. Lastly, a recently published model utilized machine-learning methods to develop a model for predicting functional independence (defined as an mRS score of 0–3) at 90 days after an ICH [29]. Factors included in their model were hematoma volume, hematoma expansion, IVH, overall ICH score, and GCS score. The c-index using a decision-tree or random forest method was 0.75 and 0.82, respectively. Our optimism-corrected c-index of 0.88 suggests including mRS score at 30 days improves the discriminative ability of the model to predict the 90-day mRS score.

Our work does have some limitations, which deserve mentioning. The population of the ATACH-2 trial were individuals who have ICH and high blood pressure and who were not receiving anticoagulation. The ATACH-2 population may not be generalizable to the general population of patients with ICH as trial populations tend to be healthier and enrolled at high volume centers. The moderate severity of the ICH, based on a median mRS score of 4, along with ATACH-2 limiting enrollment to individuals with hematoma volume less than 60 cm^3^ also limits the applicability of this model to individuals experiencing more severe or larger-volume events. External validation of our model, particularly in an anticoagulated population, would be a reasonable next step to improve the model’s generalizability. We used robust bootstrap internal validation methods to confirm model calibration and discrimination instead of a split sample derivation/validation. Unlike splitting the sample, which can reduce sample size and power [30], bootstrapping involves the entire population in both the derivation and internal validation of the model. While overfitting of the model can be of concern, our performance of shrinkage/penalization lessens concerns regarding its impact (though not eliminating them entirely). Any secondary analysis of data collected through conduction of a randomized trial can incur limitations. It is possible that our model, although performing well after internal validation, may perform poorly when externally validated in different populations for which it was not developed or intended. It is therefore critical for the model to be externally validated in targeted populations to ensure maintenance of good performance prior to being recommended for routine clinical use. Despite the mRS score having several strengths, including covering the range of functional outcomes from no symptoms to death, it also has known limitations [31]. As there is an uneven gradation of function between levels and lack of consensus for an optimal dichotomous cut point, analyses on the ordinal scale were chosen for this model. More recent focus has been on use of utility-weighted mRS score, which has potential advantages both in terms of analysis and interpretation [32]. In the present study, we found that EQ-5D weighted mRS scores were markedly similar between the predicted and observed probabilities at 90 days.

## Conclusions

The 30-day mRS score combined with baseline characteristics can reliably predict functional independence and the EQ-5D–weighted 90-day mRS in individuals with ICH. This tool allows practitioners and researchers to utilize clinically available information along with the mRS score 30 days after an ICH to predict the mRS score at 90 days.

## Supporting information

S1 Appendix Contains S1 Table (EQ-5D-3L Scores Measured at 90-Days Stratified by mRS Grouping at 90 Days), S2 Table (Differences Between Observed vs. Predicted Functional Independence (mRS Score 0–1 at 90 Days)), S3 Table (Differences Between Observed vs. Predicted Functional Independence (mRS Score 0–3 at 90 Days)), and S1 Fig (Distribution of Observed 30-day mRS Scores by Observed 90-day mRS scores.
(DOCX)

S1 Data Excel-based calculator to generate predicted 90-day mRS scores using proportional odds model. (XLSX)
