# Supplementary material for: Using 30-day modified rankin scale score to predict 90-day score in patients with intracranial hemorrhage: Derivation and validation of prediction model
Source: PLoS One. 2024 May 21;19(5):e0303757. doi: 10.1371/journal.pone.0303757 (PMC11108121; doi:10.1371/journal.pone.0303757)
Supplement: S1 Appendix — Predicted Functional Independence (mRS Score 0–1 at 90 Days)), S3 Table (Differences Between Observed vs. Predicted Functional Independence (mRS Score 0–3 at 90 Days)), and S1 Fig (Distribution of Observed 30-day mRS Scores by Observed 90-day mRS scores. (DOCX) [file pone.0303757.s001.docx]

# SUPPLEMENTAL APPENDIX

| **Content** | **Page** |
| --- | --- |
| Supplemental Table 1. EQ-5D-3L Scores Measured at 90-Days Stratified by mRS Grouping at 90 Days | 2 |
| Supplemental Table 2. Differences Between Observed vs. Predicted Functional Independence (mRS Score 0-1 at 90 Days) | 3 |
| Supplemental Table 3. Differences Between Observed vs. Predicted Functional Independence (mRS Score 0-3 at 90 Days) | 4 |
| Supplemental Fig 1. Distribution of Observed 30-day mRS Scores by Observed 90-day mRS scores^a^ | 5 |

| **90-day mRS score** | **Mean EQ-5D-3L score** |
| --- | --- |
| 0 | 0.93 ± 0.10 |
| 1 | 0.89 ± 0.11 |
| 2 | 0.79 ± 0.12 |
| 3 | 0.66 ± 0.15 |
| 4 | 0.45 ± 0.23 |
| 5 | 0.16 ± 0.11 |
| 6 | 0 |
| Total | 0.67 ± 0.26 |

EQ-5D-3L = EuroQol-5D-3L, mRS = modified Rankin Scale.

|  | | Functional independence (0-1) Predicted | | Total |
| --- | --- | --- | --- | --- |
|  |  | No | Yes |  |
| Functional independence (0-1) Observed | No | 592 | 60 | 652 |
|  | Yes | 55 | 191 | 246 |
| Total | | 647 | 251 | 898 |

mRS = modified Rankin Scale.

*Absolute agreement: 87.2%, 95%CI=84.8-89.3%

|  | | Functional independence (0-3) Predicted | | Total |
| --- | --- | --- | --- | --- |
|  |  | No | Yes |  |
| Functional independence (0-3) Observed | No | 290 | 21 | 311 |
|  | Yes | 127 | 460 | 587 |
| Total | | 417 | 481 | 898 |

mRS = modified Rankin Scale.

*Absolute agreement: 83.5%, 95%CI=80.9-85.9%


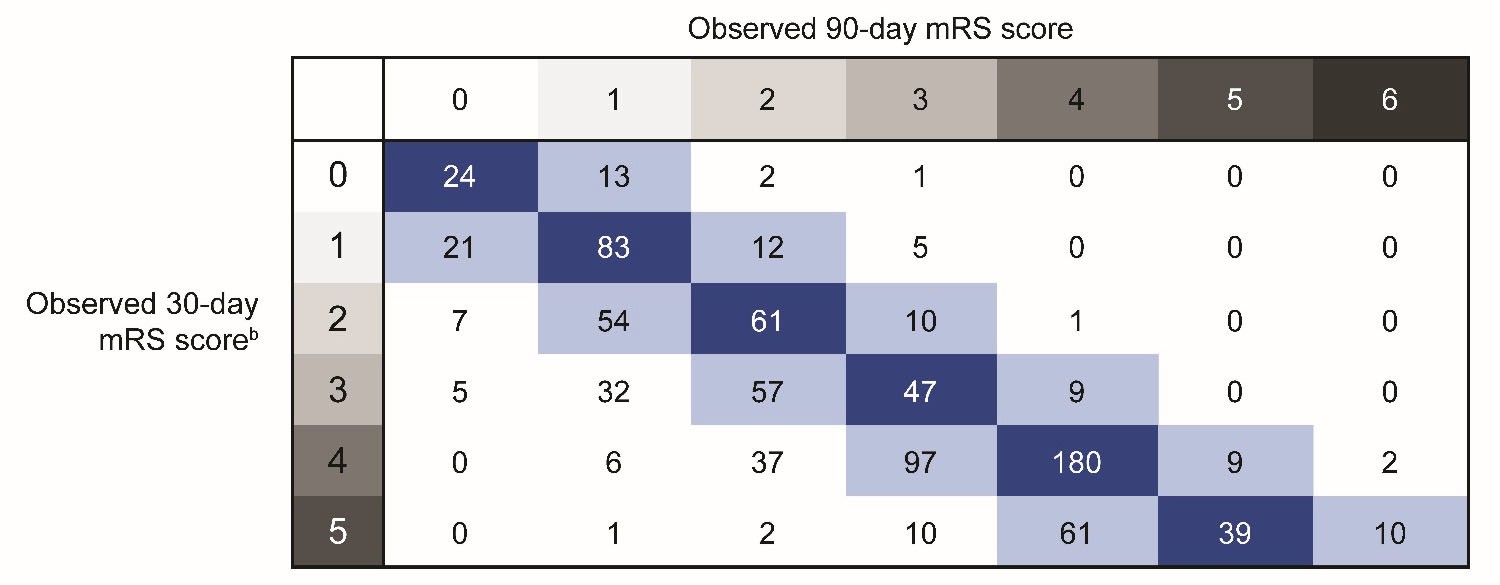


mRS = modified Rankin Scale.

^a^Any blue shading depicts agreement within 1 mRS category, dark blue shading depicts exact agreement.

^b^Patients with mRS scores of 6 at 30 days were excluded from this study.
